# Musculoskeletal MRI at 7 T: do we need more or is it more than enough?

**DOI:** 10.1186/s41747-020-00174-1

**Published:** 2020-08-06

**Authors:** Giacomo Aringhieri, Virna Zampa, Michela Tosetti

**Affiliations:** 1 Department of Translational Research and New Technologies in Medicine and Surgery, University of Pisa, Via Risorgimento, 36 Pisa, Italy; 2 Diagnostic and Interventional Radiology, University Hospital of Pisa, Via paradisa, 2, Pisa, Italy; 3 Imago 7 Foundation, Viale del Tirreno, 331 Pisa, Italy

**Keywords:** Cartilage (articular), Magnetic resonance imaging, Magnetic resonance spectroscopy, Musculoskeletal system, Ultra-high field magnetic resonance imaging

## Abstract

Ultra-high field magnetic resonance imaging (UHF-MRI) provides important diagnostic improvements in musculoskeletal imaging. The higher signal-to-noise ratio leads to higher spatial and temporal resolution which results in improved anatomic detail and higher diagnostic confidence. Several methods, such as T2, T2*, T1rho mapping, delayed gadolinium-enhanced, diffusion, chemical exchange saturation transfer, and magnetisation transfer techniques, permit a better tissue characterisation. Furthermore, UHF-MRI enables *in vivo* measurements by low-γ nuclei (^23^Na, ^31^P, ^13^C, and ^39^K) and the evaluation of different tissue metabolic pathways. European Union and Food and Drug Administration approvals for clinical imaging at UHF have been the first step towards a more routinely use of this technology, but some drawbacks are still present limiting its widespread clinical application. This review aims to provide a clinically oriented overview about the application of UHF-MRI in the different anatomical districts and tissues of musculoskeletal system and its pros and cons. Further studies are needed to consolidate the added value of the use of UHF-MRI in the routine clinical practice and promising efforts in technology development are already in progress.

## Key points

- Ultra-high field magnetic resonance imaging (UHF-MRI) provides higher spatiotemporal and spectral resolution compared to 1.5-T or 3-T MRI.
- Better morphologic, biochemical, and functional details of musculoskeletal tissues are achievable using 7-T magnets.
- UHF-MRI improves the diagnostic accuracy, in particular articular cartilage.
- Current and potential future musculoskeletal applications of UHF-MRI are rising.
- Many efforts are in progress to overcome the limitations for clinical application.

## Background

Ultra-high field (UHF) magnetic resonance imaging (MRI), in particular 7-T MRI, has a well-demonstrated role in musculoskeletal (MSK) imaging. In comparison with lower magnetic fields, UHF-MRI provides an increased signal-to-noise ratio (SNR) that can selectively increase spatial and/or temporal resolution. UHF-MRI also provides improved spectral resolution and sensitivity for X-nucleus imaging, *i.e.*, ^23^Na, ^31^P, ^13^C, and ^39^K [1]. UHF-MRI has been applied in many MSK fields, in particular for the study of cartilage but also of menisci, bone, ligaments, tendons, and skeletal muscles.

Recently, clinical interest in UHF-MRI is progressively rising, supported by the European Union and by the Food and Drug Administration (FDA) approvals for clinical 7-T MRI in 2017 [2, 3]. Most of MSK UHF-MRI reviews are mainly focused on techniques and technologies. This manuscript intends to provide a clinically oriented overview, following the pathway from the experimental settings to clinical applications, allowing the readers to more easily identify the UHF-MRI novelties for each MSK district or structure and to explore current and potential clinical applications.

## Cartilage

The main MSK field of application is cartilage, mostly regarding morphological study, ultrastructural composition, volumetric segmentation, and biochemical evaluation. The main clinical topic in cartilage studies is osteoarthritis (OA), aimed at detecting preclinical degenerative changes in ageing joints.

### Morphological imaging

The increased spatial resolution permits to evaluate microscopic morphological changes before the onset of OA symptoms, taking advantage also of shortened protocols thanks to the use of parallel imaging [4] and three-dimensional (3D) acquisitions [5]. Jin et al. [6] obtained high spatial resolution images of knee and ankle joints with an open eight-channel parallel transmission coil reaching an in-plane resolution of 0.3 mm with turbo spin-echo sequences and of 0.47 mm with isotropic dual-echo steady state acquisitions. Additionally, the U-shape coil permitted to dynamically scan the joints both in flexion and extension. Similar results were also achieved by many authors with different coils and sequences, as summarised in Table 1 [6–10].

**Table 1:** Spatial resolution obtained at 7-T

Authors [reference]	Year	Sequence type	Technique	Joint	In-plane resolution (mm)	Slice thickness (mm)
Regatte and Schweitzer [7]	2007	FLASH	3D	Knee	0.25 × 0.25	1.00
Stahl et al. [8]	2009	FIESTA-C	3D	Knee	0.25 × 0.25	1.00
Behr et al. [9]	2009	GRE	2D	Wrist, Hand	0.16 × 0.16	1.50
Behr et al. [9]	2009	GRE	3D	Wrist, Hand	0.27 × 0.27	0.30
Theysohn et al. [10]	2013	DESS	2D	Hip	0.38 × 0.38	0.76
Jin et al. [6]	2018	TSE	2D	Knee, Ankle	0.30 × 0.30	2.50
Jin et al. [6]	2018	DESS	3D	Knee, Ankle	0.47 × 0.47	0.47

*FLASH* Fast low angle shot, *FIESTA-C* Fast imaging employing steady-state acquisition with coherence interference, *GRE* Gradient-echo, *DESS* Double steady state, *TSE* Turbo spin-echo

However, all the authors underline the mandatory preliminary work on sequences optimisation and the need for dedicated coils, often not commercially available. Poor fat saturation, extensive artefacts and increased specific absorption rate (SAR) may occur during clinical protocols optimisation at UHF [8]. However, MRI technological advances will overcome these technical limitations, especially with the introduction of dedicated, multichannel, transmit-receive coils [11].

#### Knee

**Fig. 1:**
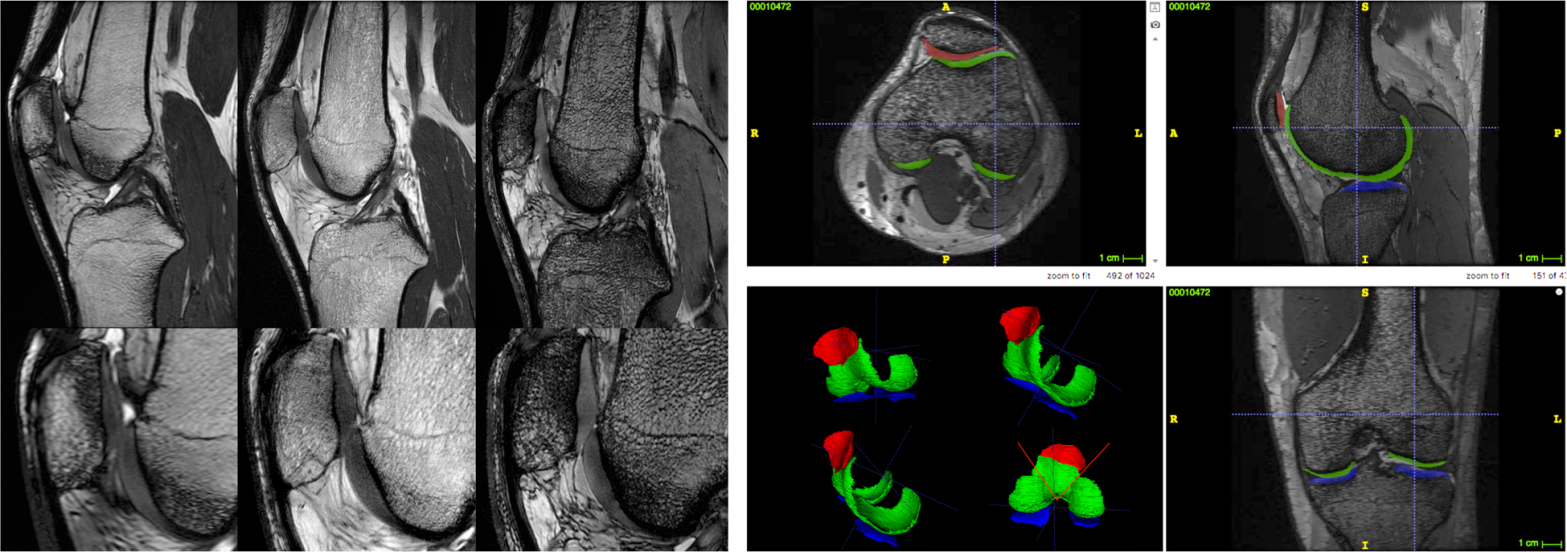
At the top left, the comparison of the same knee of the same healthy volunteer acquired with fast imaging employing steady-state acquisition with coherence interference (FIESTA-C) at 1.5-T, 3-T, and 7-T (from left to right), showing the improved spatial and contrast resolution for cartilage visualisation. At the bottom left, a detail of the comparison of the femoral-patellar compartment at 1.5-T, 3-T, and 7-T is displayed (from left to right). On the right, an example of semiautomatic segmentation of the different cartilage compartments of the knee is depicted with multiplanar reformation and corresponding volume rendering of the segmented cartilage (red, patellar cartilage; green, femoral cartilage; blue, tibial cartilage). Images were acquired at the University Hospital of Cisanello, Pisa, Italy (1.5-T and 3-T) and at the Imago7 Foundation, Pisa, Italy (7-T). Adapted with permission from Aringhieri et al, Clin Exp Rheumatol 2018. Segmentations were performed with ITK-SNAP (http://www.itksnap.org/pmwiki/pmwiki.php)

Welsch et al. demonstrated the superiority of 7-T over 3-T magnets for quantitative and qualitative cartilage evaluation, in terms of higher spatial resolution, higher contrast-to-noise ratio (CNR) and potentially reduced acquisition time [12]. Aringhieri et al. [13] showed a comparison of the overall image quality, meant as higher spatial resolution, obtained scanning the same knee of the same subject at 1.5 T, 3 T, and 7 T (Fig. 1): the optimised spatial and contrast resolution led to better cartilage depiction and boundary partial-volume artefacts reduction, enabling a more precise estimation of cartilage volumes. Springer et al. [11] analysed the diagnostic confidence comparing for the first time similar clinical protocol at 3 T and 7 T in 40 patients with knee pain. Despite a certain image quality reduction due to chemical shift artefacts, they found an improvement in overall diagnostic potential at 7 T showing an enhanced detection of subtle lesions thanks to the higher SNR and resolution.

#### Hip

Theyshon et al. [10] compared hip joints MRI at 3 T and 7 T, concluding that 7-T MRI showed slight advantages in cartilage defects and fluid detection with clinical performance comparable to 3-T magnets. However, 7-T MRI was more prone to artefacts due to B_1_ inhomogeneities, radiofrequency (RF) penetration depth and susceptibility effects around periarticular calcifications. Even magnetic fields higher than 7 T have been explored for hip MRI, as demonstrated by He et al. [14] reporting a detailed depiction of fine structures with a 0.7-mm isotropic voxel and excellent contrast at 10.5 T. Thanks to RF management strategies developed for 7-T magnets and phase shimming techniques, they obtained suitable bilateral hip images at 10.5 T with even better results when unilateral imaging was performed.

#### Ankle

Comparing ankle MRI at 3 T and 7 T, a significant increase in SNR at 7 T using a 3D gradient-echo (GRE) and two-dimensional (2D) turbo spin-echo (TSE) sequences was demonstrated, while a decrease on 2D spin-echo sequences was observed. An increased CNR was found for all the sequences except for cartilage-to-muscle contrast on the 3D GRE sequence [15]. Compared to 1.5-T MRI, 7-T MRI provided an improved depiction of the ankle anatomy and a better detection of cartilage defects and fluids, despite the usage of a non-dedicated coil. Limitations were the lower efficiency of fat saturation and reduced body coverage using TSE sequences, due to SAR limitations [16].

#### Wrist and hand

Behr et al. [9] demonstrated the superiority of 7-T over 1.5-T MRI with a better depiction of subtle anatomical details not only for cartilage but also for nerves, muscles, tendons, ligaments, pulleys, and blood vessels, especially at when studying the fingers. In particular, time-of-flight sequences resulted in a lower background signal with higher CNR, permitting detailed digital arteries visualisation, exploiting the increased T1 of tissues at 7 T. Nowadays, no studies on clinical applications of UHF-MRI in wrist and hand have been published, yet.

#### Shoulder

Lazik-Palm et al. [17] first demonstrated the feasibility of shoulder clinical protocol acquisition at 7 T with diagnostic image quality. Compared to 1.5-T MRI, higher contrast and spatial resolution obtained at 7 T led to a better image quality and morphological assessment of alterations of the articular structures, including cartilage. Nevertheless, the diagnostic performance of 7-T MRI did not result superior to the results of 3-T MRI reported in the literature [17].

### Compositional imaging: T2 mapping

The most used technique for cartilage evaluation is T2 quantitative analysis, mostly obtained as T2 mapping. Cartilage T2 depends on different extracellular matrix properties, such as tissue anisotropy, water content, collagen concentration, and integrity, while it is insensitive to proteoglycan content. Despite the decreased T2 values at UHF, many studies have been performed at 7 T providing an optimised *in vivo* representation of zonal stratification of cartilage, useful to depict early signs of OA first occurring in the superficial layer [18, 19].

**Fig. 2:**
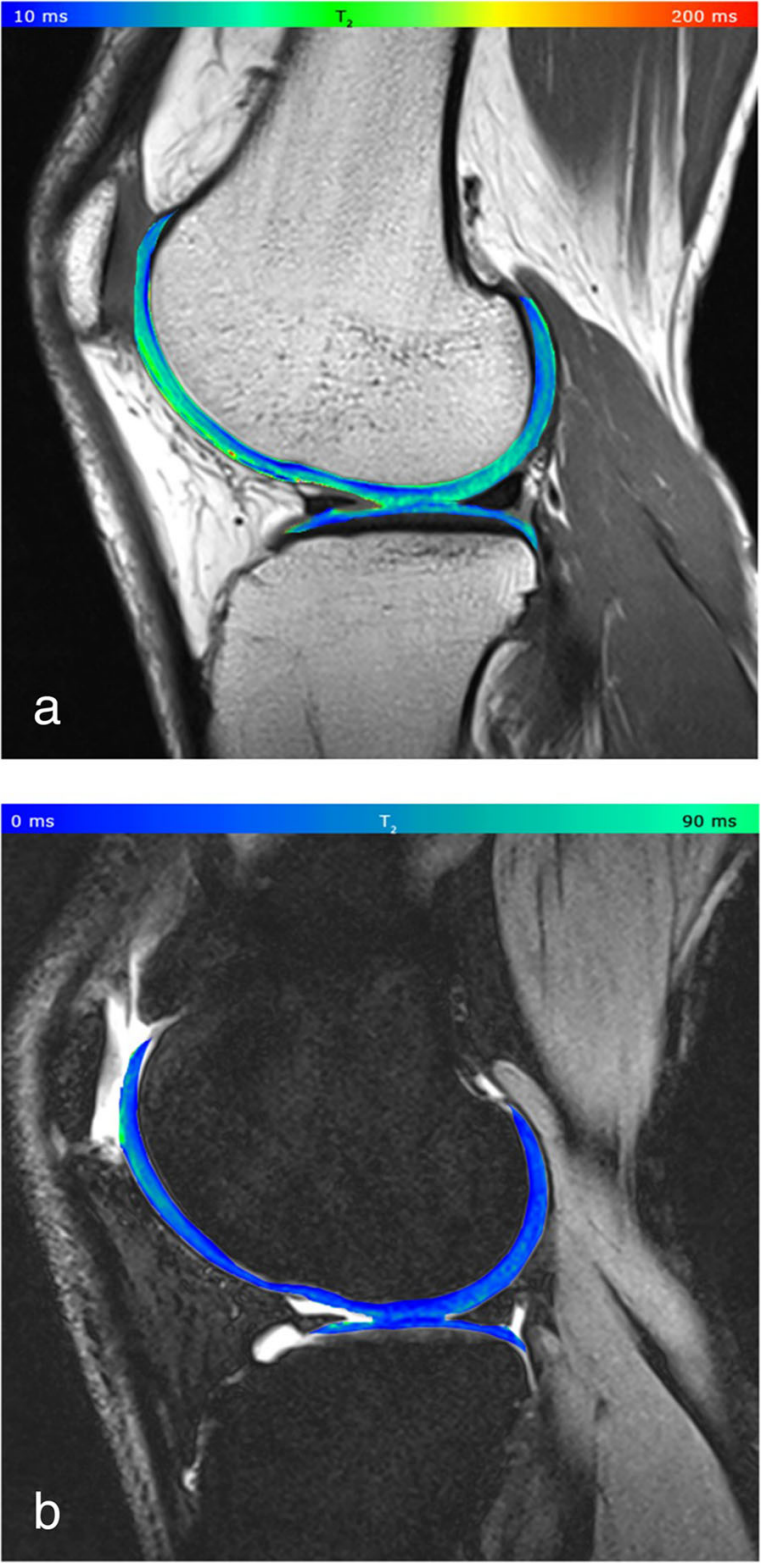
**a** Cartilage T2 map acquired with a Carr-Purcell-Meiboom-Gill (CPMG) multi-echo spin-echo (MESE) sequence, pseudo-colour-coded and overlaid on a morphological T2-weighted image from a 32-year-old healthy volunteer (echo time 11.9 ms). **b** Cartilage T2 map acquired with a three-dimensional triple echo steady state, pseudo-colour-coded and overlaid on the second acquired image (F_0_, the lowest order steady state free precession—free induction decay mode acquired in the repetition time). The typical zonal stratification of cartilage is observable with both techniques. However, the CPMG sequence provides apparently higher dynamic contrast of T2 values with better depiction of cartilaginous layers. Reprinted with permission from: Juras et al., Eur Radiol 2016 (reference [21])

**Fig. 3:**
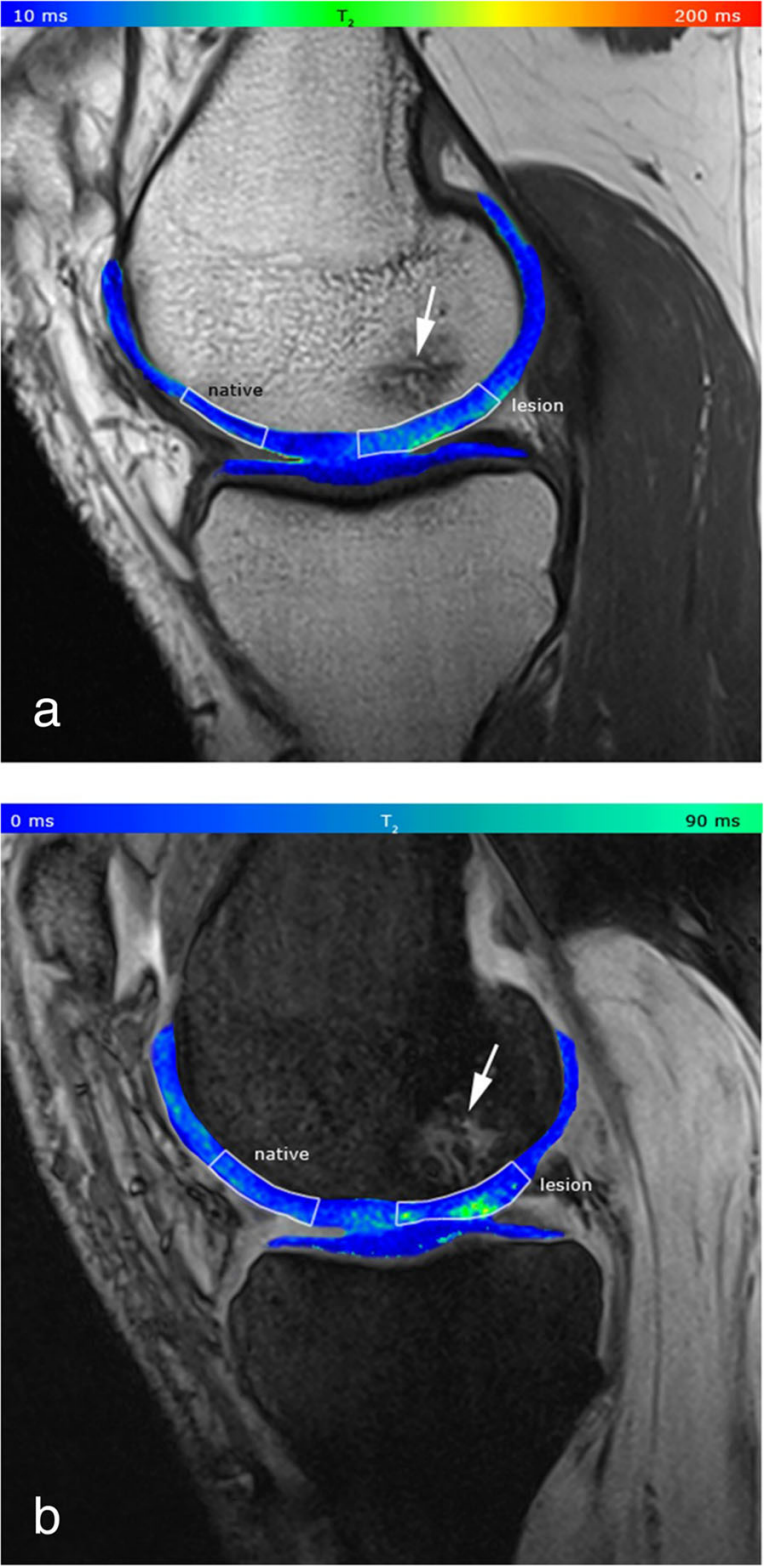
**a** T2 map acquired with Carr-Purcell-Meiboom-Gill (CPMG) multi-echo spin-echo (MESE) sequence, pseudo-colour-coded and overlaid on a morphological image of a 41-year-old patient with Pridie drilling on the medial femoral condyle (echo time 11.9 ms). The ratio between the native and lesion cartilage T2s was 0.81. **b** T2 map acquired with a three-dimensional triple-echo steady state (3D TESS) sequence, pseudo-colour-coded and overlaid on the second acquired image (F_0_, the lowest order steady state free precession—free induction decay mode acquired in the repetition time). The ratio between the native and lesion cartilage T2s was 0.83. Focal increase in T2 values is observable with both techniques. Bone oedema is also present (arrow). The dynamic range of the CMPG MESE T2 map is higher compared to that of the 3D-TESS T2 map: the lesion appears to be more diffuse, although the ratios between native and lesion cartilage T2s are comparable. Reprinted with permission from: Juras et al., Eur Radiol 2016 (reference [21])

The most used T2 mapping technique for cartilage is a multi-echo spin-echo (MESE) acquisition, which however requires long acquisition time and also implies increased SAR, induced by repeated refocusing RF pulses. Other techniques have been tested for T2 mapping, such as multiple steady state free precession-based sequences, which however are sensitive to B_1_ inhomogeneities and do not always provide an accurate and reliable T2 quantification [20]. In 2014, Heule et al. [20] introduced a novel technique, named triple echo steady state (TESS), able to acquire simultaneously T1 and T2 with no influence of B_1_ inhomogeneities and of T1 on T2, and *vice versa*. Consistently with MESE results, 3D-TESS was able to detect damaged cartilage as foci of increased T2, with a reduced scan time, insensitive to and B_0_ and B_1_ inhomogeneities. Examples of the use of this technique are given in Figs. 2 and 3.

Notably, TESS systematically provided lower cartilage T2 values compared to MESE. The overestimation of T2 values obtained with MESE technique was assumed to be related to the stimulated echo contamination due to imperfect refocusing pulses in MESE acquisitions [21, 22]. In Table 2, a summary of the mean T2 values obtained for the cartilage are displayed, showing the variations between different anatomical districts and different T2 mapping techniques [26]. When quantitative maps will be included in the clinical routine, it could be useful to perform different techniques during the same examination, including contrast-enhanced sequences. Indeed, relevant effects of gadolinium on post-contrast T2 or T2* mapping have been excluded [27].

**Table 2:** Reference T2 values of cartilage in healthy subjects

Authors [reference]	Year	Number of subjects	Age range (years)	Age (years) mean ± SD	Technique	Joint	T2 values range (ms)	T2 values (ms)mean ± SD
Welsch et al. [23]	2011	17	n.a.	25.8 ± 5.7	MESE	Knee	n.a.	41.8 ± 5.5
Domayer et al. [24]	2011	10	n.a.	30.2 ± 6.1	MESE	Ankle	n.a.	30.1 ± 4.2
Lazik et al. [25]	2016	11	21–46	n.a.	MESE	Hip	40.7–44.5	n.a.
Kraff et al. [22]	2016	8	21–39	n.a.	TESS	Hip	19.1–22.6	n.a.
Kraff et al. [22]	2016	8	21–39	n.a.	MESE	Hip	41.1–46.2	n.a.
Juras et al. [26]	2016	10	n.a.	31 ± 9	TESS	Knee	n.a.	23.5 ± 3.7
Juras et al. [26]	2016	10	n.a.	31 ± 9	TESS	Ankle	n.a.	18.8 ± 2.6

Reference T2 values of the normal cartilage acquired with different techniques in different joints. As reported by Juras et al. [26], in addition to differences in cartilage structure and signal within the same anatomical district, T2 differences are present between different joints, reflecting the different structural composition (Source: Juras et al., Eur J Radiol 2016 - reference [26]). *MESE* Multi-echo spin-echo sequence, *n.a.* Not available, *SD* Standard deviation, *TESS* Triple-echo steady state

#### Knee

Welsch et al. [28] first demonstrated significantly increased T2 values at 7 T, from the deep to the superficial layer of the cartilage, in all the knee compartments, reflecting the different orientation of the collagen fibres: perpendicular to the subchondral bone in the deep zone and parallel to the chondral surface in the superficial zone, as previously demonstrated by Smith et al. [29]. Compared to 3 T, at 7 T a higher CNR was observed and, despite the absolute T2 reduction, a zonal differentiation was still detectable albeit less pronounced [23]. Post-surgical UHF T2 mapping showed significant increased T2 in cartilage repair tissues compared to native cartilage with no significant difference between superficial and deep layers [30, 31]. Among the different biochemical features, only T2 values had significant correlation with the clinical outcome [31]. Wyatt et al. [32] analysed T2 values obtained with 3D magnetisation-prepared angle-modulated partitioned k-space acquisition in healthy volunteers and patients with OA comparing 3-T with 7-T MRI. T2 mapping at 7 T allowed to detect degenerate cartilage in the knee with results comparable to 3-T MRI. Indeed, 7-T MRI provided more significant differences in T2 values between healthy and degenerate cartilage compared to 3 T.

Recently, Juras et al. [19] prospectively evaluated patients with low-grade cartilage lesions at 3 T and and 7 T, demonstrating that 3-T T2 mapping is more sensitive for low-grade lesions than 7-T T2 mapping due to a higher short component of T2, that is considerably reduced at 7 T.

#### Hip

Despite the complex anatomy and the thin layer of cartilage, feasibility of 7-T T2 mapping in the hip was demonstrated by Lazik-Palm et al. [25]. T2 maps with MESE were obtained with minor artefacts, such as pulsation artefacts due to inguinal vessels and inhomogeneities in the dorsal part of the hip. Kraff et al. [22] demonstrated that TESS at 7 T improves quantitative hip cartilage imaging with insensitivity to B_1_ inhomogeneities, shortening scan time and comparable quality and repeatability compared to MESE. Feasibility of T2 mapping after cartilage transplant has been demonstrated by Lazik-Palm et al [33], comparing 3-T to 7-T MRI. Despite an apparent overestimation of T2 values at 7 T compared to 3 T, the intra-individual ratio between T2 values of cartilage transplant and normal acetabular and femoral cartilage obtained at 7 T were comparable to that obtained at 3 T, confirming the accuracy of UHF T2 mapping in post-surgical evaluation of the hip [33].

#### Ankle

**Fig. 4:**
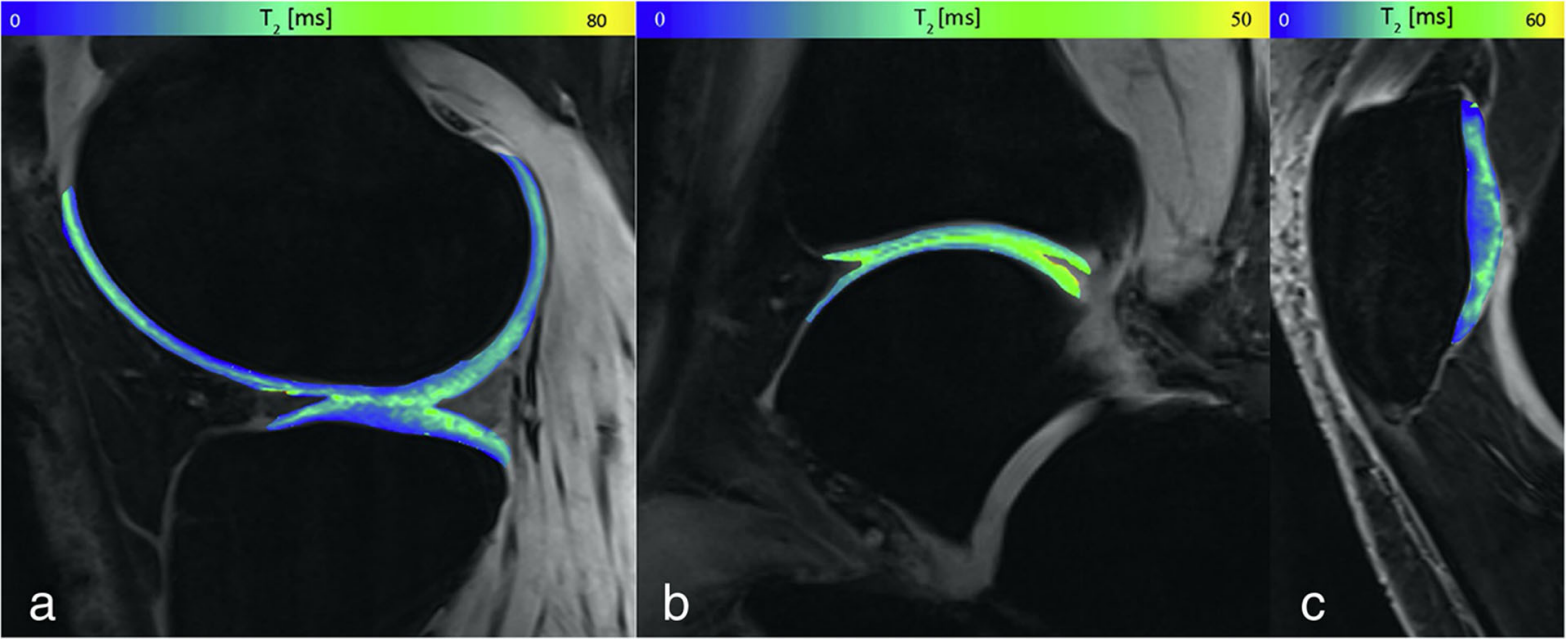
T2 maps of cartilage of the knee (**a**), ankle (**b**), and patella (**c**) of pseudo-colour-coded three-dimensional triple echo steady state three-dimensional triple-echo steady state overlaid on the second acquired image (F_0_, the lowest order steady state free precession - free induction decay mode acquired in the repetition time). Reprinted with permission from: Juras et al, Eur J Radiol 2016 (reference [26])

As for the hip, similar challenges are present for ankle imaging. Domayer et al. [24] demonstrated MESE T2 mapping feasibility, finding a significant zonal differentiation between superficial and deep layer with higher T2 values in the first one. In repair cartilage tissues after surgical treatments, T2 values in the superficial layers were similar to regular cartilage; the deep layers showed significantly increased T2 values, reflecting the abnormal increased water content and lower collagen and glycosaminoglycan (GAG) concentration in reparative tissues. Juras et al. [26] successfully tested 3D TESS T2 mapping in the ankle, finding significant differences of T2 values in the ankle compared to the knee (Fig. 4; see Table 2).

### Compositional imaging: T2* mapping

T2* relaxation time reflects the different properties of cartilage extracellular matrix and is obtained with multi-echo GRE T2*-weighted sequences, with at least six echo times. As for T2, T2* shows a zonal differentiation increasing from the deep to the superficial layer, with a larger gap of T2* compared to T2. This effect reflects the susceptibility fields induced by the cartilage-to-bone interface with T2* shortening [28]. Compared to MESE T2 mapping, T2* mapping allows 3D acquisition and shorter acquisition times, due to a reduced repetition time. However, this technique is more prone to susceptibility artefacts (which are greater at 7 T) that could affect the precision of T2* quantification, especially in case of periarticular calcification and subchondral sclerosis. Compared to 3-T MRI, 7-T MRI showed a reduced knee cartilage T2* values with a less pronounced zonal differentiation between deep and superficial layer [23].

### Compositional imaging: T1rho mapping

Spin-lattice relaxation time in the rotating frame (T1rho) mapping reflects proteoglycan (PG) and collagen cartilage content and has been tested at 7 T in knee and hip joints [25, 32]. In damaged cartilage, characterised by a decreased content of PG, T1rho values tend to increase due to the altered spine-lattice exchanges between protons and the environment. In particular, even if no changes were observed in T2 mapping, slight decrease of PG content was observed with T1rho technique, allowing to detect subtle initial degenerative changes in ageing cartilage [34]. Singh et al. [35] obtained high-SNR and high-resolution 3D T1rho mapping of the knee cartilage at 7 T with an in-plane resolution of 0.2 mm^2^. Compared to 3 T, at 7 T they reached the same resolution in a four-fold reduced scanning time.

Wyatt et al. [32] compared T1rho mapping of the knee in healthy volunteers and patients with OA at 3 T and 7 T and found increased T1rho values in the patellar cartilage in patient with OA at both field strengths. In the lateral femoral condyle, the same results were obtained at 3 T but were not observed at 7 T. However, many cartilage regions approached significance at UHF and all the *p* values were lower than those obtained at 3 T, confirming UHF as a promising tool despite the need for further studies to consolidate its superiority.

### Compositional imaging: delayed gadolinium-enhanced MRI of cartilage

The first MRI contrast medium introduced in clinical practice is gadopentetate dimeglumine (Gd-DTPA^2-^). Due to its anion form, it is repelled by the negatively charged GAGs and, thus, it is used to estimate GAGs content in the cartilage. Areas of damaged cartilage, with reduced GAGs content, tend to accumulate Gd-DTPA^2-^, resulting in shorter T1 values.

Welsch et al. [28] demonstrated delayed gadolinium-enhanced MRI of cartilage (dGEMRIC) *in vivo* feasibility at 7 T in the knee with T1 mapping acquisitions in healthy volunteers before and 90 min after intravenous Gd-DTPA^2-^ administration, asking the subjects to exercise the knee for 20 min. Although T1 values at 7 T were lower compared to those obtained at lower field strengths, the use of pre-/post-contrast ratio represents a promising tool to estimate GAGs content in native and repair cartilage.

Potential future introduction of this technique in clinical practice will benefit from a reduction in scanning time, which can be obtained omitting the pre-contrast T1 mapping acquisition. Indeed, post-contrast T1 values were demonstrated sufficient to assess the cartilage health status in the hip at 7 T thanks to its linear correlation with the difference in relaxation rate between pre-contrast and post-contrast T1 values [25].

Despite the high specificity for GAGs, long scanning time, need for post-injection exercise and intravenous contrast medium administration limit the dGEMRIC technique, which result in being more invasive and expensive than others. Moreover, evidences for gadolinium retention in the brain (and in other human tissues) have been published, although no clinically or biologically relevant consequences have been demonstrated, yet [36].

### Compositional imaging: diffusion tensor imaging

Since different components of the cartilage matrix affect the motion of water molecules, diffusion tensor imaging can provide information about PG content and collagen architecture. PGs lead to a motion restriction in all the directions, affecting the mean diffusivity. Collagen network induces anisotropy in the water motion, which can be measured with fractional anisotropy [37]. Although diffusion techniques are challenging at UHF, Raya et al. [38] succeeded in patellar cartilage evaluation at 7 T using line-scan DT imaging. It is inherently insensitive to motion artefacts and B_1_, B_0_ and susceptibility inhomogeneity; the poor SNR was overcome by using UHF MRI and multi-channel coils. Matrix alterations led to increased apparent diffusion coefficient (ADC) and reduced fractional anisotropy, permitting to significantly differentiate damaged from healthy cartilage. Damaged cartilage showed increased ADC values both in the superficial and in the deep layers, showing a promising role of ADC as early indicator of cartilage degeneration.

### Compositional imaging: magnetisation transfer

In cartilage, there are two proton pools in biochemical and magnetic equilibrium: free (unbound) water protons, visible by MRI, and macromolecules-bound protons, not visible by MRI. When bound protons are saturated by off-resonance pulses, the equilibrium shifts in favour of bound protons, reducing the detectable magnetisation and the signal. Therefore, magnetisation transfer acquisition, based on steady state free precession sequences, can provide quantitative biochemical cartilage evaluation [30]. At 7 T, knee cartilage magnetisation transfer showed significantly reduced mean values compared to 3 T with a significant zonal stratification detectable only at 7 T [23].

### Compositional imaging: chemical exchange saturation transfer

GagCEST method estimates the cartilage GAG content by selective saturation of protons within the hydroxyl groups of cartilage GAGs, reducing the signal intensity due to the magnetisation exchange with free water. UHF MRI improves the gagCEST performance for the increased SNR as well as for the more selective saturation between hydroxyl GAG and water protons due to the increased gap in resonance frequencies [18, 38–40].

The first gagCEST application at 7 T was performed for post-surgical knee cartilage evaluation [41]. GagCEST feasibility and its role as a potential imaging biomarker of cartilage GAG content was proven, based on its correlation with the high-GAG-specific sodium imaging [41]. A 3 T *versus* 7 T comparison study stated the need for UHF MRI to accurately estimate GAGs avoiding direct saturation effects and fast exchange rate observed at 3 T [42]. Regarding post-surgical long-term cartilage evaluation, a significant GAGs reduction in repair cartilage compared to native cartilage was demonstrated, albeit without significant correlation with the clinical outcome [31].

Promising results have been obtained by applying modified technical parameters to selectively optimise the chemical exchange saturation transfer (CEST) signal from hydroxyl protons of exogenous hyaluronic acids (HAs) to evaluate their distribution in the cartilage (viscoCEST). Compared to viscoCEST, gagCEST detected signal also from exogenous GAGs, while viscoCEST was not able to depict endogenous GAGs. This could represent a promising tool to track the intraarticular distribution of hyaluronic acid after viscosupplementation [43].

### Compositional imaging: sodium

The negatively charged lateral chains of GAG within the cartilage attract sodium atoms, making sodium signal an imaging biomarker for the cartilage matrix. UHF MRI performed with dedicated coils permits to partially overcome the 3,000 times lower SNR of sodium compared to proton [18].

Wang et al. [44] first obtained 3D sodium images of the whole knee at 7 T in less than 15 min with a 3D GRE sequence with radial k-space acquisition. They observed a significant reduction in sodium concentration in patients with OA compared to healthy subjects. Recently, a two-fold reduced scan time was obtained for 7-T MRI of the knee by introducing compressed sensing techniques without losing accuracy in total sodium concentration for detecting early signs of OA [45].

**Fig. 5:**
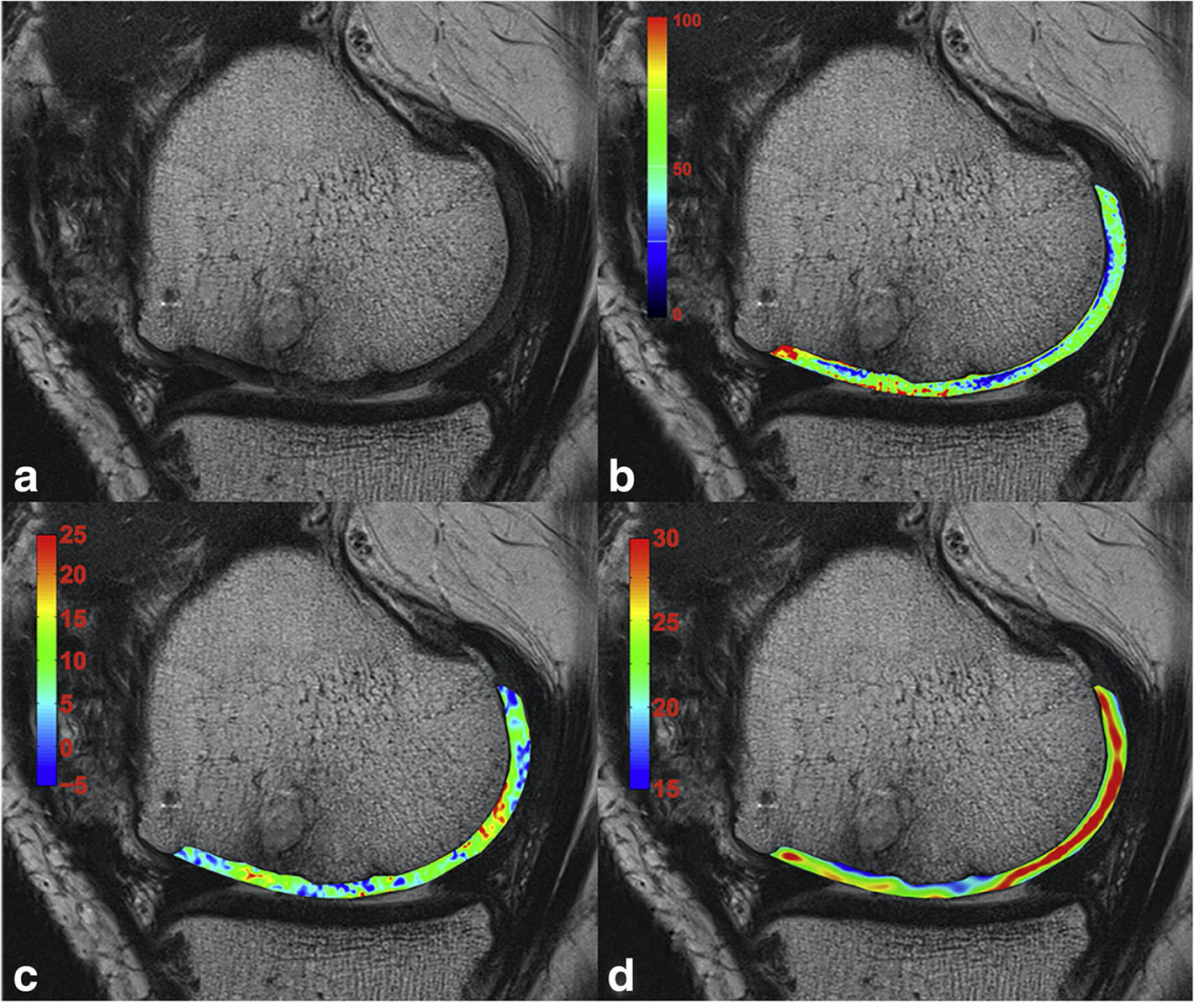
7-T MRI of a patient (mean age of subjects included: 49 years old; interquartile range, 44–55) 8 years after osteochondral treatment at the medial femoral condyle. **a** Morphologic proton-density fast spin-echo image. **b** Graphical overlay with T2 map. Colour bar represents relaxation times (ms) (higher values, more water and disturbed collagen architecture). **c** Graphical overlay with glycosaminoglycan chemical exchange saturation transfer (gagCEST) image. Colour bar represents gagCEST asymmetries in % (lower values, less proteoglycan content). **d** Graphical overlay with sodium image. Colour bar represents the sodium signal-to-noise ratio values (lower values, less proteoglycan content). Reprinted with permission from: Krusche-Mandl et al., Osteoarthr Cartil 2012 (reference [31])

The low sensitivity of sodium imaging requires dedicated and optimised coils; dual tuned coils (^1^H/^23^Na) have been tested to reduce the reciprocal induced degradation of the sensitivity. Although many progresses have been made, we do not have the optimal coil yet and further efforts are still needed [46, 47]. Moreover, quantitative sodium imaging is affected by partial volume effects including articular fluid. Different inversion recovery techniques have been proved to overcome this challenge, albeit the increase in SAR [48, 49]. For its role in early damaged cartilage evaluation, sodium imaging has been tested for the evaluation of different post-surgical cartilage repair tissues. A significantly lower sodium signal in repair cartilage tissue compared to native cartilage was observed (Fig. 5). Despite a difference in GAG content in cartilage repair tissues after surgical treatments, no significant difference for the clinical outcomes among the different surgical approaches was demonstrated [31, 50].

## Bone

Many studies have been performed focusing on trabecular bone quantitative evaluation in osteoporosis and OA. Mechanical properties of the trabecular bone depend not only on the bone volume fraction (density) but also on the internal arrangement. While usual clinical techniques, such as dual-energy x-ray absorptiometry, evaluate bone density alone, novel methods called micro-finite element (micro-FE) techniques estimate every aspect of bone internal architecture through reconstruction and modelling of high-resolution 3D datasets. So far, these datasets could be given by micro-computed tomography, limiting this approach to small size bone/bone samples [51]. Chang et al. [52] demonstrated the feasibility of micro-FE of the knee providing high-resolution 3D datasets with 3D fast low angle shot acquisitions at 7 T. With a 7-min scan, they could differentiate contributions of trabecular and cortical bone to the whole bone stiffness. Trabecular bone resulted predominant in long bones epiphyseal stiffness thus representing a better target for treatments in reducing fractures. Combining UHF MRI and micro-FE could help to evaluate bone mechanical competence in osteoporosis and, focusing on subchondral bone, its role in OA pathogenesis.

Regarding 7-T quantitative bone imaging applications, Chang et al. [53] demonstrated an improvement in trabecular bone architecture in response to elevated mechanical stress in athletes.

Krug et al. [54] compared balanced steady state free precession and balanced steady state spin-echo techniques for trabecular bone estimation at 3 T and 7 T. The number of measured trabeculae was more accurate at UHF, albeit some overestimation of trabecular volume occurred due to the increased susceptibility effects. A bigger potential for UHF balanced steady state spin-echo technique was suggested due to the reduced off-resonance artefacts which cause trabecular broadening and small trabeculae disappearance.

Preliminary data of 7-T *ex vivo* studies demonstrated feasibility of ultrashort time of echo (UTE) T2* mapping of cortical bone with high-resolution isotropic acquisition [55] and suggested potential applications in the spine [56]. Finally, Li et al. [57] demonstrated feasibility of 7-T MRI of bone marrow perfusion of the distal femur without intravenous administration of contrast agent using the arterial spin labelling approach, although further technical developments are needed.

## Ligaments and tendons

Conventional MRI visualisation of tendons and ligaments can be difficult due to the low signal given by their short T2 [58]. Among the novel sequences, UTE has been demonstrated useful to better depict these structures [59], including the fascicular pattern in the Achilles tendon with stripes of higher signal representing endotendon and inner areas of lower signal representing fascicles. Similarly, in the knee, different structures like tendons, ligaments and menisci were depicted in less than 5 min with 0.7–0.8-mm isotropic resolution [60].

**Fig. 6:**
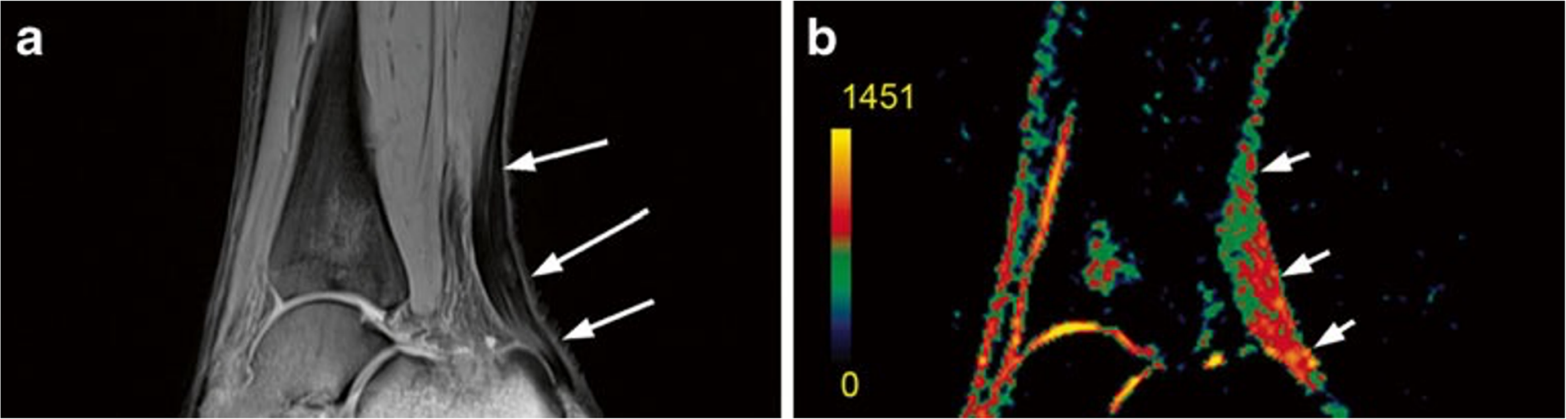
MRI of a 46-year-old patient with chronic Achilles tendinopathy. **a** Proton-density-weighted two-dimensional turbo spin-echo image depicts centres of each region of interest (arrows). **b** Corresponding colour-coded three-dimensional gradient-echo sodium image depicts Achilles tendon sodium signal (arrows), which is obviously higher than that in the healthy volunteer. The sodium signal increase was observed in the whole tendon, and not only in regions with clinical findings (middle region in this case). All patients had focal thickening of the Achilles tendon without fluid-like signal intensity increase on proton-density–weighted two-dimensional turbo spin-echo images. Colour scale represents sodium signal intensity values. Reprinted with permission from: Juras et al., Radiology 2012 (reference [63])

Juras et al. [61] compared 3-T and 7-T 3D UTE T2* mapping of the Achilles tendon in volunteers and in patients with tendinopathy. Interestingly, SNR was higher at 3 T while the contrast was almost double at 7 T. A significant increase in T2* values in patients compared to healthy volunteers was observed. Since T2* mapping resulted sensitive for biochemical changes related to tendinopathy, it could represent a useful imaging biomarker for early detection of tendon degeneration. The same technique with fat saturation led to a better microstructure visualisation of the Achilles tendon with improved SNR at 7 T compared to 3 T [62]. Regarding sodium imaging, Juras et al. [63] demonstrated a significant increase in sodium signal in patients with tendinopathy compared to healthy volunteers (Fig. 6), reflecting the increase in GAG content. The authors found an increased amount of GAGs not only in the area of tendinopathy depicted by morphological imaging but also in the whole tendon, with a possible role in detecting patients at risk for Achilles tendon tear [63].

A comparison between 7-T MRI and arthroscopy of the shoulder has been performed by Lazik-Palm et al. [17], demonstrating the feasibility of clinical protocol acquisition at 7 T with diagnostic image quality in patients with suspected tendinopathy of the rotator cuff. Compared to the arthroscopic evaluation, 7-T MRI led to an overestimation of tendinopathy, especially regarding supraspinatus and subscapularis tendons. This was explained by the authors, on the one hand, with the interpretation bias due to the magic angle effect, which creates artefactual hyperintensities in structures orientated at 55° to the main magnetic field, even if reduced compared to lower field strengths. On the other hand, the expected higher resolution of 7-T imaging and the patient selection criteria might have led to a misinterpretation of the signal alterations [17]. At the state of the art, the diagnostic accuracy of 3-T MRI still remains superior to 7-T MRI of the shoulder [17] and further studies are needed in order to include UHF-MRI of the shoulder in clinical practice.

Concerning ligaments, Anz et al. [64] compared 7-T and 3-T quantitative evaluation of T2/T2* values and volumes of the anterior cruciate ligament in the knee of 30 healthy volunteers (Table 3). An accurate quantitative evaluation of the anterior cruciate ligament might represent a promising tool to assess the “*ligamentisation*” process of tendon grafts after surgery. Despite these results, no demonstrable advantage in 7-T MRI over 3-T MRI was observed.

**Table 3:** Anterior cruciate ligament quantitative MRI at 3 T and 7 T

Field strength	T2 values (ms) Mean ± SD	T2* values (ms) Mean ± SD	Volume (mm^3^) Mean ± SD
3 T	37.1 ± 7.9	10.9 ± 1.3	2,380 ± 602
7 T	39.7 ± 6.2	10.9 ± 0.9	2,484 ± 736

Source: Anz et al., Skeletal Radiol 2020–reference [64]. *SD* Standard deviation

## Menisci and intervertebral discs

As for other connective tissues, meniscal MRI visualisation benefits from UHF: higher SNR, higher resolution and better contrast at 7 T enable a more accurate detection of subtle morphological meniscal alterations compared to clinical field strengths [7]. UHF MRI also allows T2* quantification of the meniscal ultrastructure with a demonstrated relationship between T2* decay and collagen fibres orientation. Although further studies are needed to transfer this method into the clinical practice, these results might help in the interpretation of T2 and T2* quantitative images of early meniscal degeneration at clinical field strengths with lower resolution [65]. Important information on disc ultrastructure and degeneration was obtained *ex vivo* with a correlation between matrix degeneration and distribution of PG diffusivity and PG CEST. Thus, ADC and CEST values have been proposed as imaging biomarkers of early disc degeneration. Moreover, PG T2 values correlated only with severe disc degeneration [66].

Feasibility of disc sodium imaging and T2 mapping *in vivo* at 7 T was first demonstrated by Noebauer-Huhmann et al. [67], who performed a comparison with a semiquantitative morphological score for intervertebral disc degeneration (Pfirrmann score [68]) in healthy volunteers. Sodium imaging and T2 maps values showed a positive correlation and a moderate negative correlation with the Pfirrmann score respectively, while no correlation was found between sodium and T2 values. This work paved the way for future *in vivo* applications in the disc degeneration assessment.

## Muscles

Muscles can be evaluated by MRI in terms of structural and functional imaging. Despite the improved SNR and the higher resolution, no morphological UHF studies have been performed in muscles, yet. Integrating 7-T images with data from computed tomography, Kerkhof et al. [69] created an *ex vivo* complete 3D highly accurate virtual musculoskeletal model of forearm and hand, including relevant physiological parameters. This open-source study provides a free 3D anatomical model useful for further studies in physio-pathological musculoskeletal research.

Many studies confirmed UHF advantages for magnetic resonance spectroscopy (MRS) due to the improved spectral resolution and the capability of X-nuclei imaging [70]. Among the different nuclei, phosphorus (^31^P) is the main target of UHF evaluation of muscles. In particular, many papers focused on inorganic phosphorus and phosphorylated metabolites estimation to assess mitochondrial function in skeletal muscles recovery. Kan et al. [71] first published data obtained with direct *in vivo* quantification of the inorganic phosphate pool (P_i_) in resting muscles in healthy subjects at 7 T. The transmit and receive dual-tuned (^1^H/^31^P) coil, the high SNR, the improved spectral and spatial resolution permitted to differentiate cytosolic P_i_ from mitochondrial P_i_. At the same time, signals from phosphorylated muscle metabolites, usually observable at lower field strengths, were also detectable at 7 T.

Subsequent works confirmed the gain in spectral, temporal and spatial resolution obtained at 7 T for ^31^P imaging to investigate muscle recovery with different techniques at rest and during exercise [72, 73]. Multiple phosphate metabolites as well as the single metabolite estimation can be performed with different techniques, with consequent influence on the total scan time [72].

Recently, quantitative detailed dynamic evaluation of muscles metabolism has been demonstrated to be feasible thanks to the higher SNR provided by dedicated coils, efficient localisation and UHF. Additionally, cytosolic buffer capacity and H^+^ efflux from muscle cells to blood in early recovery were non-invasively quantified for the first time [73].

**Fig. 7:**
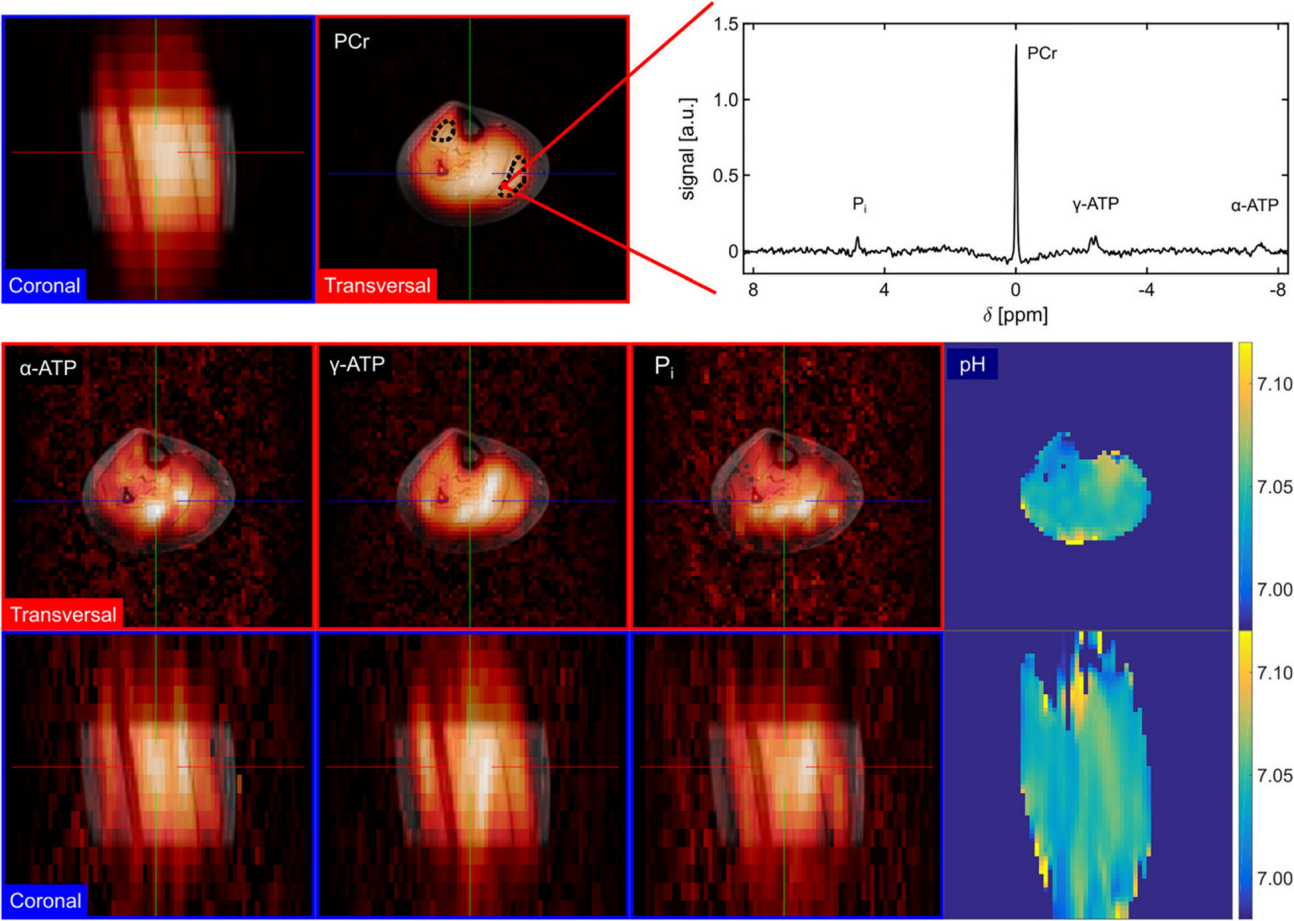
Three-dimensional *in vivo*^31^P–{^1^H} echo planar spectroscopic imaging dataset from the human calf muscle. The shown ^31^P–{^1^H} spectrum was localised in a voxel inside the medial gastrocnemius muscle (indicated by bold red line; effective size 4.05 mL). Transversal and coronal metabolic maps superimposed on ^1^H images of Phosphocreatine (PCr), α- and γ-adenosine triphosphate (ATP), inorganic phosphate (Pi), and pH are displayed. The position of transversal and coronal slices is indicated by the thin red and blue lines, respectively. Absolute intensity scales of α- and γ-ATP, Pi are cropped for better visualisation. The PCr maps were used to create a mask for the corresponding pH maps. Regions for evaluation of signal-to-noise and metabolite ratios are illustrated by the black dashed lines. For the measurement parameters and the acquisition strategy see the reference article’s text. Reprinted with permission from: Korzowski et al., Magn Reson Med 2018 (reference [74])

Last updates on ^31^P imaging demonstrated feasibility of high-resolution ^31^P imaging performed with multishot 3D echo planar spectroscopic imaging instead of the more used chemical shift imaging. This novel approach permitted to obtain high-resolution 3D mapping of ^31^P-metabolites and intracellular pH reducing the scan time up to 10 min with a voxel size of 4 cm^3^ (Fig. 7) [74].

To indirectly estimate creatine (Cr) concentration, CrCEST technique exploits the chemical exchange saturation transfer effect between its amine and bulk water protons. In addition to the good agreement between CrCEST and ^31^P MRS in the recovery kinetics after exercise, CrCEST provides a three-fold sensitivity enhancement compared to ^31^P MRS [75]. Recently, Kogan et al. [76] demonstrated that CrCEST in the lower leg muscles of healthy volunteers at 7 T is not influenced by blood flow during recovery.

Several promising techniques to non-invasively investigate the different characteristics of skeletal muscles were tested at UHF, including potassium MRS for intracellular evaluation [77] and ^13^Carbon MRS for absolute quantitative assessment of intramuscular glycogen in storage diseases [78]. Finally, Towse et al. [79] compared the blood oxygenation-level dependence technique in healthy volunteers, demonstrating a significant increase in CNR and SNR in the evaluation of muscles microcirculation at 7 T compared to 3 T.

## Limitations

While specific limitations of each technique are discussed above, general limitations that could affect image quality at UHF are B_1_ and B_0_ inhomogeneities, increased T1 relaxation times and decreased T2 relaxation times, which require mandatory time-consuming preliminary optimisation. Increased susceptibility and chemical shift, exploited for specific diagnostic purposes, also lead to artefacts. At UHF, higher RFs and shorter wavelengths may result in decreased penetration with inhomogeneous excitation. Indeed, these also cause increased SAR [38]. MRI below 8 T is considered safe by FDA; nevertheless, SAR limits are established [80]. While global SAR limits are usually respected in MSK UHF MRI, attention must be paid to potential local SAR increase.

Different prediction methods for SAR spatial distribution, including machine learning, have been tested to prevent potential health risks for patients [81, 82].

Although an increase in physiologic side effects have been reported at UHF (dizziness, nausea and metallic taste) [83, 84], patients’ experience seems to be similar to that perceived at lower field strengths [85].

Finally, the high costs for installation and maintenance of UHF MRI scanners and their low availability actually represent a not negligible limitation for their routine clinical use.

## Future perspectives and conclusions

Future perspectives will depend on the constantly evolving innovation in hardware/software and sequences, such as implementation of parallel imaging [6, 86], B_1_ shimming techniques [87] and MRI fingerprinting [88]. Another fascinating novel application is represented by early detection of synovitis in rheumatoid arthritis, even though still tested in animal models [89]. As in other fields [90, 91], staging of bone tumours in children could be a future potential application of UHF MRI, exploiting the high spatial resolution to better define the growth plate invasion with potentially less invasive surgical treatments and improved quality of life. The higher SNR could also provide a more accurate visualisation of vessels both with and without intravenous administration of contrast agent [92].

In conclusion, UHF MRI provides important diagnostic improvements in the field of MSK imaging. Higher SNR and CNR permits to obtain higher spatiotemporal resolution with improved anatomic detail of the musculoskeletal structures and higher diagnostic confidence. Additionally, improvements in spectroscopic and quantitative imaging provide biochemical and metabolic information allowing a better tissue characterisation. Approvals for clinical imaging at UHF were the first step towards a routine use of this technology. However, studies are needed to consolidate the advantages of a routine clinical utilisation of UHF-MRI. To overcome its limitations, promising efforts in hardware and software development are already in progress.

## Data Availability

Not applicable.

## Abbreviations

ADC: Apparent diffusion coefficient
CEST: Chemical exchange saturation transfer
CNR: Contrast-to-noise ratio
Cr: Creatine
FDA: Food and Drugs Administration
GAGs: Glycosaminoglycans
Gd-DTPA: Gadopentetate dimeglumine
GRE: Gradient echo
MESE: Multi-echo spin-echo
Micro-FE: Micro-finite element
MRI: Magnetic resonance imaging
MRS: Magnetic resonance spectroscopy
MSK: Musculoskeletal
OA: Osteoarthritis
PG: Proteoglycan
Pi: Inorganic phosphate pool
RF: Radiofrequency
SAR: Specific absorption rate
SNR: Signal-to-noise ratio
T1rho: Spin-lattice relaxation time in the rotating frame
TESS: Triple echo steady state
TSE: Turbo spin-echo
UHF: Ultra-high field
UTE: Ultrashort time of echo
